# Modulated Photocurrent Spectroscopy for Determination of Electron and Hole Mobilities in Working Organic Solar Cells

**DOI:** 10.1038/s41598-019-56945-3

**Published:** 2019-12-30

**Authors:** Hiroki Nojima, Takashi Kobayashi, Takashi Nagase, Hiroyoshi Naito

**Affiliations:** 1 Department of Physics and Electronics, Osaka Prefecture University, 1-1 Gakuen-cho Naka-ku, Sakai, 599-8531 Japan; 2 The Research Institute for Molecular Electronic Devices (RIMED), Osaka Prefecture University, 1-1 Gakuen-cho Naka-ku, Sakai, 599-8531 Japan

**Keywords:** Electrical and electronic engineering, Electronic devices, Optical spectroscopy, Semiconductors, Photovoltaics

## Abstract

Carrier drift mobility is an important physical constant in the charge transport process of organic solar cells (OSCs). Although time-of-flight and space-charge-limited current techniques have been frequently utilized for mobility measurements, the validity of a new method using modulation photocurrent spectroscopy is discussed in this contribution. The advantages of this method are its applicability to working OSCs with optimized device structures and the simultaneous determination of the electron and hole mobilities. These features make it possible to study the relation between the mobility balance and the solar cell characteristics, such as the power conversion efficiency, using only a single working OSC; hence, it is not necessary to fabricate electron-only and hole-only devices for mobility measurements. After carrying out numerical simulations to examine the validity of this method for mobility determination, the dependence of the mobility balance on the mixing ratio of the electron-donor and –acceptor materials is presented.

## Introduction

Organic solar cells (OSCs) with bulk-heterojunction (BHJ) structures have several advantages, such as flexibility, light weight, and low-cost production, with respect to inorganic counterparts and so they are expected to be the main power source for flexible and wearable devices^1–3^. So far enormous efforts have been made to improve their power conversion efficiency (PCE) as well as to understand the operational mechanisms. It is known that carriers photogenerated in BHJ layers travel due to the electric field originating from the built-in potential and are ideally collected by a pair of the electrodes without being lost to a recombination process. However, if either electron or hole drift mobility is low, space charge will be formed in the BHJ layer and the recombination loss significantly reduces the carrier collection efficiency^4–6^. To achieve a high PCE, therefore, it is essential to increase the electron and hole drift mobilities while keeping them in balance. The strong correlation between the mobility balance and PCE in OSCs has been pointed out by numerical simulations carried out by Kotlarki *et al*.^6^. The mobility of organic semiconductors is known to depend on the thermal annealing temperature^7–9^, the solvent used for spin-coating^10^, and the mixing ratio of electron-donor and -acceptor materials^11–16^. Therefore, while using a given combination of the donor and acceptor materials, a good mobility balance may be achieved by tuning the sample preparation conditions. In fact, after the report by Kotlarki *et al*., the correlation between a mobility balance and the solar cell characteristics has been investigated experimentally^17–26^ and the tendency that a good mobility balance results in a high PCE was confirmed^21,24–26^. In these investigations, however, the mobility was measured with the space-charge-limited current (SCLC) technique. The disadvantage of the SCLC^5,7,11,16^ and the time-of-flight (TOF) techniques^8,14^, the latter being also frequently used for mobility measurements, is that cells with improper metal electrodes (for SCLC) and intentionally thick BHJ layers (for TOF) have to be prepared separately from the cells for the PCE evaluations. This means that in those publications, the PCE and the mobility were evaluated using cells with different device structures. Although there have been some other alternative experimental techniques^9,12,13,27,28^ used for mobility measurements, it is difficult to simultaneously determine both the electron and hole mobilities in working OSCs.

**Figure 1:**
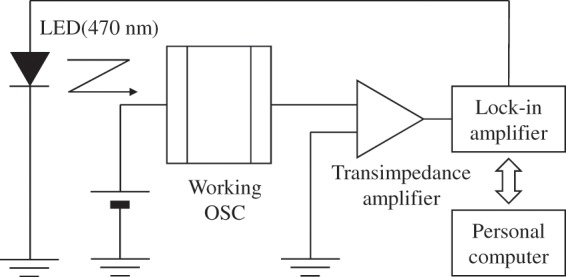
Block diagram of the setup for the MPC measurements.

In this report, we demonstrate that the electron and hole mobilities in working BHJ OSCs can be determined with a modulated photocurrent (MPC) technique, which is one of the modulation spectroscopic techniques and can be carried out with a relatively simple experimental setup consisting of a sinusoidally modulated light source and a lock-in amplifier (see Fig. 1)^29^. The MPC technique was originally developed in 1950’s as a method to study localized-state distributions in amorphous inorganic^30–35^ and organic^36^ semiconductors, and has been recently applied to BHJ OSCs to investigate charge transport processes such as interface recombination and charge lifetime^37–42^. As shown below, the MPC technique can also measure the time period from the photocarrier generation to their arrival to either electrode. However, there is no report that the mobility in organic semiconductors can be determined from the time period (referred to as the transit time hereafter) that is measured with the MPC technique. In this report, we first show the numerical simulations that were carried out to clarify the relation between the transit time and the mobility in the dispersive and non-dispersive conduction modes. The results demonstrate an additional advantage of MPC with respect to the TOF technique, that is, a single analytical method can be used to determine the mobility regardless of the conduction mode. We also present the experimental results from BHJ OSCs based on poly(3-hexylthiopehene-2,5-diyl) (P3HT) and [6,6]-phenyl-C_61_-butyric acid methyl ester (PCBM) including the correlation between the measured mobilities and PCE.

## Results

### Numerical simulations of MPC measurements

**Figure 2:**
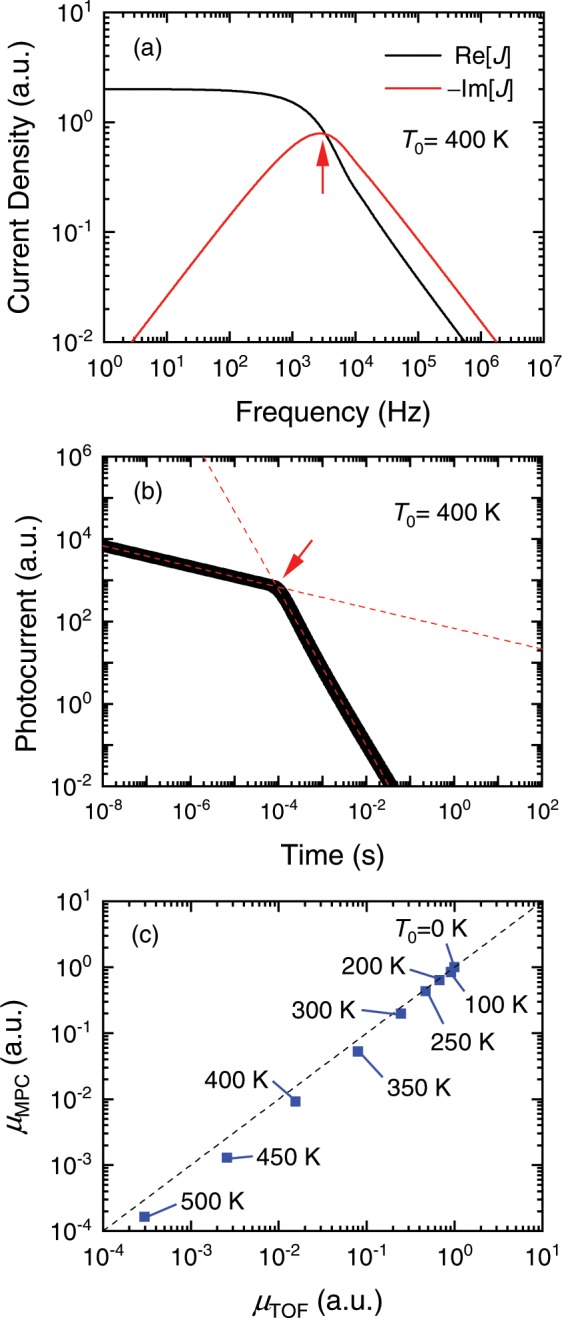
(**a**) Simulated modulation frequency dependence of the real and imaginary parts of the modulated photocurrent. (**b**) Simulated temporal evolution of the photocurrent. The dashed lines are guides to the eye. The results in panel (**a**,**b**) are obtained from the same set of the physical constants including *T = *300 K and *T*_0_ = 400 K. The red arrows indicate the frequency used to calculate the transit time in panel (**a**) and the transit time itself in panel (**b**). (**c**) Comparison between the mobilities determined with the MPC and TOF techniques from the photocurrent responses simulated with various *T*_0_’s. In the last panel, 0 < *T*_0_ < 300 K corresponds to the non-dispersive conduction mode, and the dashed line represents a slope of 1.

In organic semiconductors, charge transport processes are significantly influenced by localized states. This is evidenced by the dispersive photocurrent transients recorded in TOF measurements^43–45^. Such photocurrent transients are simulated by solving the continuity equation considering localized states with an exponential distribution^46,47^. Also, results of the MPC measurements, e.g., the modulation frequency dependence of the photocurrent, can be simulated by the same equations under small-signal sinusoidal photoexcitation^35,48,49^. For the details of the simulations, see the Supplementary Information. An example of the simulated results is shown in Fig. 2(a). In modulation spectroscopy, not only the amplitude of the photocurrent (*J*) but also its phase delay are important. One way to properly express the modulation frequency (*f*) dependence of the amplitude and the phase delay is to plot in-phase and out-of-phase components of *J* as a function of *f*. The in-phase and out-of-phase components are also referred to as the real and imaginary parts of *J*, or simply Re[*J*] and Im[*J*], respectively. The simple Debye dielectric function tells us that the out-of-phase component has its peak at the frequency (*f*_peak_) that is related to the characteristic relaxation time of a medium^50^. This relaxation time corresponds to the transit time (τ_t_) of the photocarriers for BHJ OPVs under a short circuit condition. As shown in the Supplementary Information, τ_t_ is related to *f*_peak_ as

1 $${\tau }_{t}=\frac{1}{4{f}_{peak}},$$

under the assumption that the photocarriers are uniformly excited throughout the BHJ layer. From this assumption, the drift mobility μ is given by

2 $$\mu =\frac{L}{2{\tau }_{t}F},$$

where *F* is the applied electric field and *L* is the thickness of the BHJ layer. Throughout this work, we determine τ_t_ and μ with Eqs. (1) and (2). In Fig. 2(b), we also show the transient *J* simulated with the same set of the physical constants, and the transit time is measured from the time where the slope of the transient *J* in a double logarithmic plot changes. This way to measure the transit time is frequently used for an analysis of the dispersive transients^43,46,51,52^. We carried out the simulations for a room temperature (*T* = 300 K) with several characteristic temperatures (*T*_0_’s) of the exponential distribution of the localized states and compared the determined mobilities [see Fig. 2(c)]. In the case of the non-dispersive conduction mode (*T*_0_ < 300 K), the transit time is uniquely defined and is accurately measured with the MPC and TOF techniques. Thus, the μ values determined with these techniques completely coincide with each other. On the other hand, in the case of the dispersive conduction mode (*T*_0_ > 300 K), the transit time may be defined in several ways. Since the approaches used to measure the transit time in the MPC and TOF techniques are not exactly equivalent, the two determined μ values are gradually separated from each other as *T*_0_ increases. For example, when *T*_0_ = 400 K^47^, the μ value from the MPC technique is almost half of that using TOF. Within this error, however, the same μ values can be obtained with the MPC and TOF techniques.

### Experimental measurements

P3HT and PCBM blends are one of the combinations of the donor and acceptor materials that have been extensively investigated for OSC applications. The electron and hole field-effect mobilities of this combination have been reported to depend on the mixing ratio^13,14,16^. Solar cell characteristics of the fabricated devices based on this combination are summarized in Table 1 and their current density-voltage characteristics are shown in Fig. S2 in the Supplementary Information. Among the fabricated devices, the best PCE of 3.5% was obtained for the P3HT concentration of 60 wt% and *L* = 200 ± 10 nm. When the P3HT concentration differs from the optimum value, the fill factor (FF) and the PCE degrade.

**Table 1:** Photovoltaic characteristics including the open circuit voltage *V*_*OC*_ and the short circuit current density *J*_*SC*_ of the fabricated OSCs.

P3HT concentration (wt%)	*V*_*OC*_ (V)	*J*_*SC*_ (mA/cm^2^)	FF	PCE (%)
70	0.58	6.8	0.55	2.2
60	0.59	8.6	0.69	3.5
50	0.58	9.3	0.56	3.0
40	0.57	6.5	0.52	1.9
30	0.55	3.2	0.48	0.84

**Figure 3:**
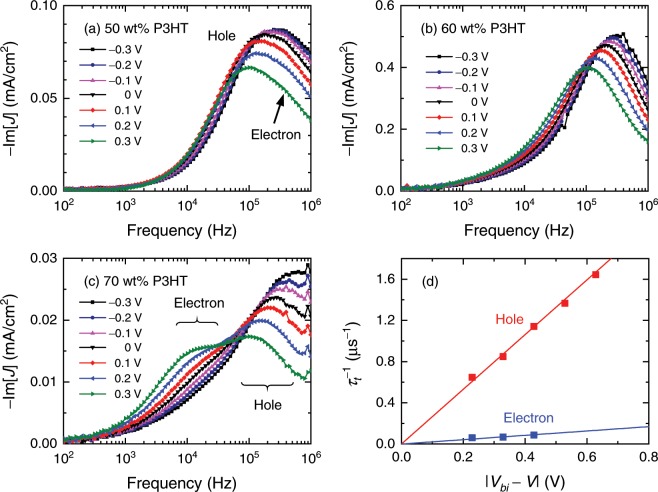
–Im[*J*]-*f* characteristics of OSCs with the P3HT contents of (**a**) 50, (**b**) 60, and (**c**) 70 wt% under various bias conditions. Reverse biases are expressed as negative values. (**d**) The inverse of the determined transit times versus the effective applied bias for the OSC with the 70 wt% P3HT content [panel (**c**)]. The solid lines are the linear fits.

**Figure 4:**
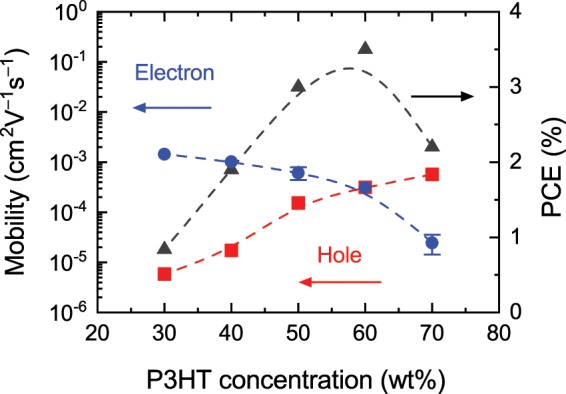
P3HT concentration dependence of the determined electron and hole mobilities and measured PCE. The dashed lines are guides to the eye (b-spline curves). The error bars indicate the degree of uncertainty of the peak frequencies determined from the shoulders.

We applied the MPC technique to such devices under various bias conditions. The resultant −Im[*J*]-*f* characteristics of OSCs with the P3HT concentrations of 50, 60 (the optimum), and 70 wt% are shown in Fig. 3(a–c), respectively. While two peaks are clearly seen in Fig. 3(c), only one peak is observed in Fig. 3(b). In Fig. 3(a), the second peak can be found around 300 kHz as a small shoulder. These peaks can be attributed to the transit times of the photocarriers because the peaks shift to the higher frequency side as the applied reverse bias increases and also as *L* is reduced. The fact that the photocurrent signals are remarkably suppressed at the P3HT concentration of 100 wt% (not shown here) indicates that the peaks observed in Fig. 3(a–c) are due to the photocarriers that are generated at the interface between P3HT and PCBM. In Fig. 3(d), we plot the reciprocal of the transit times obtained from Fig. 3(c) as a function of the effective bias, which is the difference between the applied voltage *V* and the built-in potential *V*_bi_. As expected from Eq. (2), both the shorter and longer transit times are directly proportional to the effective bias. From the slopes, the two mobilities are determined to be 5.7 × 10^−4^ and 2.5 × 10^−5^ cm^2^V^−1^s^−1^. All the mobilities determined in this work are summarized in Fig. 4, in which higher and lower mobilities seem to be switched at the 60 wt% P3HT concentration. In a BHJ layer with a low P3HT (PCBM) concentration, insufficient permeation networks for hole (electron) transport are formed and thus result in the degradation of the hole (electron) drift mobility^11,12^. Therefore, the lower and higher mobilities at lower P3HT concentrations can be attributed to holes and electrons, respectively. The highest hole and electron drift mobilities determined are in good agreement with the reported values for P3HT and PCBM neat thin films, respectively, measured with TOF or SCLC techniques^8,14,16,53–55^. From Fig. 4, it is also found that the electron and hole mobilities are well balanced at the optimum (60 wt%) P3HT concentration, where the best PCE is realized. It should be noted that the PCE is dependent not only on the mobility balance but also on other factors, e.g., the absorption spectrum. It is thus expected that the FF has a more straightforward relation with the mobility balance than the PCE. Such a correlation between the FF and the mobility balance is indeed observed experimentally^21,24–26^. Therefore, the best PCE in this work should be mainly attributed to the highest FF.

## Discussion

**Figure 5:**
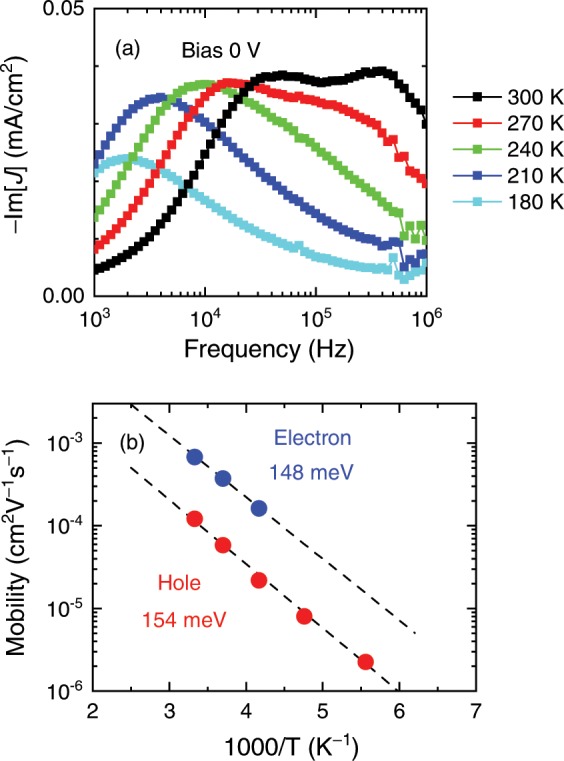
(**a**) –Im[*J*]-*f* characteristics of OSCs with a 56 wt% P3HT content measured at different temperatures under no applied bias. (**b**) Temperature dependence of the determined mobility. The dashed lines are the best fits with Eq. (3).

If only one peak is observed in a –Im[*J*]-*f* characteristic, it cannot be determined whether the transit times of electrons and holes coincide, or either of them is out of the measurable frequency range. One way to find a missing peak is to compare several –Im[*J*]-*f* characteristics for different donor concentrations. The peaks that are completely overlapped at a particular donor concentration would be separated from each other at another concentration, as demonstrated above. This approach also makes it possible to know which mobility is that for electrons. Another way to find a missing peak is to record the –Im[*J*]-*f* characteristics at different temperatures. If the activation energies of the donor and acceptor materials are different, the two peaks in the –Im[*J*]-*f* characteristic would shift to the lower frequency side with different rates at lower temperatures. An example of the MPC measurements at different temperatures is shown in Fig. 5(a), in which two peaks are recognized at a room temperature but one disappears at lower temperatures. The mobilities determined from the peak frequencies are plotted in Fig. 5(b). Since the measured temperature range is narrow, we assume an Arrhenius-type temperature dependence and fit the following equation to the hole mobilities:

3 $$\mu ={\mu }_{0}{\rm{e}}{\rm{x}}{\rm{p}}(-\frac{{E}_{a}}{{k}_{B}T}),$$

where *k*_*B*_ is the Boltzmann’s constant, μ_0_ is the mobility at *T = *∞ K, and *E*_*a*_ is the activation energy. From the fits, *E*_*a*_ is determined to be 154 meV for holes and 144 meV for electrons. The former is consistent with the *E*_*a*_ values (124–160 meV) measured on P3HT neat thin films with the SCLC technique^56,57^. The latter value is also between the *E*_*a*_ values determined in field-effect transistors based on pure PCBM (112 meV) and a P3HT:PCBM blend with 66 wt% P3HT concentration (160 meV)^58^. Fig. 5(b) shows the possibility that the overlapped –Im[*J*] peaks can indeed be separated from each other at a different temperature. Another merit of lowering the temperature is that a peak that is above the measurable frequency range at room temperature may be shifted lower within the range at lower temperatures; this means that the measurable range is expanded^49,59^.

## Conclusions

In conclusion, we have demonstrated that the MPC technique is a powerful tool to determine the carrier drift mobility of BHJ OSCs. Its advantages with respect to other methods, such as TOF and SCLC techniques, are its applicability to a working OSC with an optimum structure and simultaneous determination of electron and hole mobilities. Since no special cells are needed for the mobility determination, the relation between PCE and the mobilities can be investigated. As an example of such an investigation, we demonstrated that a good mobility balance (*μ*_e_ = *μ*_h_ = 3.1 × 10^−4^ cm^2^V^−1^s^−1^) that is achieved by tuning the mixing ratio of P3HT and PCBM results in the best PCE of 3.5% within the OSCs fabricated in this work.

## Methods

### Solar cell fabrication and characterization

Inverted OSCs with an effective area of 4 mm^2^ were fabricated on indium tin oxide (ITO)-coated glass substrates with a 2 mm stripe pattern. The device structure was ITO/ZnO/P3HT:PCBM (200 ± 10 nm)/MoO_3_ (10 nm)/Al (50 nm). The BHJ layer was spin-coated from chlorobenzene solutions containing P3HT and PCBM at a spin-rate of 800 rpm. The mixing ratio of P3HT and PCBM was varied while the concentration of the solutions was kept constant so that the resultant BHJ layer thickness stayed almost the same. The weight ratio of P3HT to the mixed solute was changed from 30 to 80 wt%. All the fabrication procedures were done in a glove box filled with nitrogen gas, and the OSCs were taken from the glove box after the encapsulation. The current density-voltage characteristics were recorded with a source meter under 100 mW/cm^2^ AM1.5G irradiation.

### MPC measurements

The MPC measurements were carried out with a home-made setup consisting of a LED with 470 nm emission, a lock-in amplifier, and a transimpedance amplifier. Modulated LED light was irradiated from the ITO side of an OSC. Various biases were applied to an OSC with a DC power supply. During the measurements at lower temperatures, an OSC was set in a vacuum chamber attached to a nitrogen bath, a heater, and a temperature controller. The OSC was maintained in a vacuum of 10^−4^ Pa and within a temperature range from 150 to 300 K.

### Numerical simulations

The details of the numerical simulations are described in the Supplementary Information. All the numerical simulations were performed using Microsoft, Visual Studio (C + + ) and NAG, Fortran Builder.

## Supplementary information

Supplementary information
