# Supplementary material for: Modulated Photocurrent Spectroscopy for Determination of Electron and Hole Mobilities in Working Organic Solar Cells
Source: Sci Rep. 2019 Dec 30;9:20346. doi: 10.1038/s41598-019-56945-3 (PMC6937248; doi:10.1038/s41598-019-56945-3)
Supplement: Supplementary file 1 — Supplementary information [file 41598_2019_56945_MOESM1_ESM.pdf]

Supplementary Information

Modulated Photocurrent Spectroscopy for Determination of Carrier Drift Mobility in Working Organic Solar Cells

Hiroki Nojima[1], Takashi Kobayashi[1,2], Takashi Nagase[1,2], and Hiroyoshi Naito[1,2]

[1]Department of Physics and Electronics, Osaka Prefecture University, 1-1 Gakuen-cho Naka-ku, Sakai, 599-8531, Japan.

[2]The Research Institute for Molecular Electronic Devices (RIMED), Osaka Prefecture University, 1-1 Gakuen-cho Naka-ku, Sakai, 599-8531, Japan.

This supplementary information includes the details of the numerical simulations for MPC measurements and the current-voltage characteristics of the fabricated OSCs.

S1. Numerical simulations

Charge transport in a semiconducting material is described with a current-continuity equation. Initially, we consider only electrons, which are traveling in a single active layer sandwiched with a pair of electrodes. We assume that the localized states density g at an energy E is given by

$$g(E) = g_0 \exp\left(-\frac{E_C - E}{k_B T_0}\right),$$

where g_0 is the localized state density at the conduction band edge E_C, k_B is the Boltzmann's constant, and T_0 is the characteristic temperature. The continuous distribution of the localized states is split into closely spaced, discrete levels with an energy width ΔE (see Fig. S1). Trapping and detrapping rates (w_j and r_j, respectively) of the jth discrete level are written as

$$w_j = cg(E)\Delta E, \qquad (1)$$

$$r_j = \nu \exp\left(-\frac{E_C - E}{k_\mathrm{B} T}\right), \qquad (2)$$

where c is the capture coefficient and ν is the attempt-to-escape frequency. Using the free electron density n and Eqs. (1) and (2), the time evolution of the electron density trapped at the jth level is described by

$$\frac{\partial n_j}{\partial t} = w_j n - r_j n_j. \qquad (3)$$

Then, the one-dimensional current-continuity equation for n is written as

$$\mu_0 F \frac{\partial n(x,t)}{\partial x} + \frac{\partial n(x,t)}{\partial t} + \sum_{j=1}^{m} \frac{\partial n_j(x,t)}{\partial t} = G(x,t), \qquad (4)$$

where μ_0 is the free-carrier mobility, F is the electric field, and x is the charge transport direction. In this equation, G represents the electron generation due to the sinusoidally modulated light and is represented by

$$G(x,t) = G_0 + G_1 \exp(i\omega t), \qquad (5)$$

where G_0 and G_1 are the dc and ac generation rates, ω is an angular frequency, and i is the imaginary unit. Under the small-signal condition, the free and trapped electron densities can be written in the form

$$n(x,t) = n_0(x) + n_1(x) \exp(i\omega t), \qquad (6)$$

$$n_j(x,t) = n_{j0}(x) + n_{j1}(x) \exp(i\omega t). \qquad (7)$$

By substituting Eqs. (5)-(7) into Eq. (4), the following solution can be found

$$n_1(x) = \frac{G_1}{\mu_0 F} \exp\left[-\frac{i\omega x}{\mu_0 F} \left(1 + \sum_{j=1}^{m} \frac{w_j}{r_j + i\omega} \right) \right].$$

Thus, the ac photocurrent $J_1(\omega)$ can be calculated as follows

$$J_1(\omega) = \frac{q\mu_0 F}{L} \int_0^L n_1(x)\, dx \qquad (8),$$

where q is the elementary charge and L is the active layer thickness. In the trap-free condition, the peak of $-\mathrm{Im}[J_1(\omega)]$ appears at $f = \mu_0 F (2L)^{1}$. This relation can be analytically obtained from

$$\frac{\partial}{\partial \omega} \mathrm{Im}[J_1(\omega)] = 0.$$

If the transit time is defined as $\tau_t = L/(2\mu_0 F)^{1}$, we obtain $f = (4\tau_t)^{1}$. Although we consider only the electrons so far, the total photocurrent becomes the sum of the electron and hole currents as long as recombination processes in the BHJ layer are negligible. In the OSCs under a short circuit condition, the recombination processes are usually minor; this can be experimentally confirmed from the photoexcitation independence of the peak frequency.

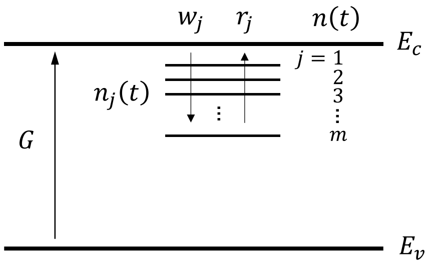

Figure S1. Localized states represented by discrete energy levels. In this figure, E_c and E_v are the energies of the conduction and valence band edges, respectively.

The modulation frequency dependence of the real and imaginary parts of the modulated photocurrent in Fig. 2(a) in the main text was simulated with Eq. (8). On the other hand, the transient photocurrent in Fig. 2(b) was obtained by solving Eqs. (3) and (4) with the Laplace-transform technique[1,2]. For these simulations, the physical constants listed in Table S1 were used.

Table S1. Physical constants used for the simulations in the main text [Fig. 2(a) and (b))]

Physical constants	
G_1	1×10^{20} cm^{-3}s^{-1}
T	300 K
L	1×10^{-5} cm
μ_0	1×10^{20} cm^{2}V^{-1}s^{-1}
V	0.65 V
c	1×10^{-8} cm^{3}s^{-1}
ν	1×10^{12} Hz
g_0	1×10^{21} cm^{-3}eV

S2. Current density-voltage characteristics of the fabricated OSCs

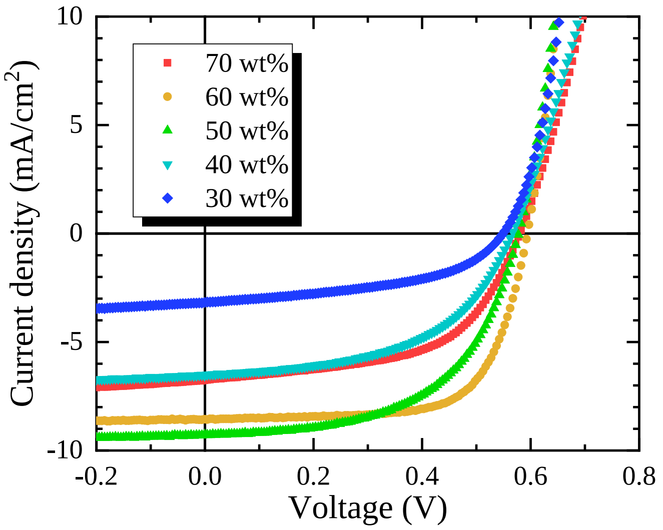

Figure S2. Current density-voltage characteristics of OSCs with various P3HT concentrations. These current-voltage characteristics were measured with a source meter under 100 mW/cm^2 AM1.5G irradiation.

References

[1] Schmidlin, F. W. Theory of trap-controlled transient photoconduction. *Phys. Rev. B* **16**, 2362-2385 (1977).

[2] Meyer, H., Haarer, D., Naarmann, H. & Hörhold, H. H. Trap distribution for charge carriers in poly(paraphenylene vinylene) (PPV) and its substituted derivative DPOP-PPV. *Phys. Rev. B* **52**, 2587-2598 (1995).
